# The Value of Enhanced MR Radiomics in Estimating the IDH1 Genotype in High-Grade Gliomas

**DOI:** 10.1155/2020/4630218

**Published:** 2020-10-24

**Authors:** Lei Niu, Wei-hua Feng, Chong-feng Duan, Ying-chao Liu, Ji-hua Liu, Xue-jun Liu

**Affiliations:** ^1^Radiology Department, The Affiliated Hospital of Qingdao University, Qingdao 266000, China; ^2^Neurosurgery Department, Shandong Provincial Hospital Affiliated to Shandong First Medical University, Jinan, China 250021

## Abstract

**Background:**

The prognosis of IDH1-mutant glioma is significantly better than that of wild-type glioma, and the preoperative identification of IDH mutations in glioma is essential for the formulation of surgical procedures and prognostic assessment.

**Purpose:**

To explore the value of a radiomic model based on preoperative-enhanced MR images in the assessment of the IDH1 genotype in high-grade glioma.

**Materials and Methods:**

A retrospective analysis was performed on 182 patients with high-grade glioma confirmed by surgical pathology between December 2012 and January 2019 in our hospital with complete preoperative brain-enhanced MR images, including 79 patients with an IDH1 mutation (45 patients with WHO grade III and 34 patients with WHO grade IV) and 103 patients with wild-type IDH1 (33 patients with WHO grade III and 70 patients with WHO grade IV). Patients were divided into a primary dataset and a validation dataset at a ratio of 7 : 3 using a stratified random sampling; radiomic features were extracted using A.K. (Analysis Kit, GE Healthcare) software and were initially reduced using the Kruskal-Wallis and Spearman analyses. Lasso was finally conducted to obtain the optimized subset of the feature to build the radiomic model, and the model was then tested with cross-validation. ROC (receiver operating characteristic curve) analysis was performed to evaluate the performance of the model.

**Results:**

The radiomic model showed good discrimination in both the primary dataset (AUC = 0.87, 95% CI: 0.754 to 0.855, ACC = 0.798, sensitivity = 85.5%, specificity = 75.4%, positive predictive value = 0.734, and negative predictive value = 0.867) and the validation dataset (AUC = 0.86, 95% CI: 0.690 to 0.913, ACC = 0.789, sensitivity = 91.3%, specificity = 69.0%, positive predictive value = 0.700, and negative predictive value = 0.909).

**Conclusion:**

The radiomic model, based on the preoperative-enhanced MR, can effectively predict the IDH1 genotype in high-grade glioma.

## 1. Introduction

Glioma is one of the most common primary malignant tumors in the brain and one of the most lethal malignant tumors in humans. An accurate preoperative diagnosis and definite characterization are very important for the selection of surgical options. In 2008, Parsons et al. [1], for the first time, found a mutation in isocitrate dehydrogenase-1 (IDH1) in the study of glioblastoma. Subsequently, a large number of studies showed significant differences in prognosis between IDH1-mutant and wild-type gliomas. The prognosis of IDH1-mutant glioma is significantly better than that of wild-type glioma [2–9]. However, at present, conventional imaging and functional magnetic resonance imaging (such as DWI, ASL, and DCE) could not predict the glioma genotypes before surgery and could not evaluate the glioma genotypes before clinical surgery. Only through the results of immunohistochemistry after surgery can we classify the tumor genes and judge the effect of surgery, predict the prognosis of patients, and even fine tune the later treatment plan. Therefore, an accurate preoperative assessment of the IDH1 genotype is of great significance for clinical treatment and prognosis evaluation.

As a new research method, radiomics can assess the phenotypes of tumors that cannot be seen by the naked eye and carry out objective and quantitative analyses to excavate the internal information of tumors and quantitatively analyze tumor heterogeneity. At present, radiomics has been verified that it can effectively improve the diagnostic accuracy [10] and has been widely used in the study of multiple organ diseases in humans.

In this study, we investigated the value of radiomics in predicting the IDH1 genotype of high-grade glioma by studying the radiomic prediction model of preoperative-enhanced MR images.

## 2. Materials and Methods

### 2.1. Patient Selection

The data were collected from our institution, a university-affiliated hospital. An approval from the institutional review board was obtained, and a written informed consent was waived due to the retrospective nature. Between December 2012 and January 2019, a total of 182 glioma patients were reviewed, and all tumors were confirmed by a histopathological examination. The subjects included 79 patients with an IDH1 mutation (45 patients with WHO grade III and 34 patients with WHO grade IV; age range, 24-62 years; mean age, 44 ± 11 years; gender, 49 males and 30 females) and 103 patients with wild-type IDH1 (33 patients with WHO grade III and 70 patients with WHO grade IV; age range, 9-78 years; mean age, 49 ± 16 years; gender, 38 males and 65 females). A total of 182 glioma patients were divided into the primary dataset (mutant IDH1, 55; wild-type IDH1, 72) and the validation dataset (mutant IDH1, 24; wild-type IDH1, 31) at a ratio of 7 : 3 using a stratified random sampling.

The inclusion criteria of this study were as follows: (1) all lesions were histopathologically confirmed by surgery, and an immunohistochemical analysis was performed, which included determining the IDH1 genotype (mutant/wild-type). All patients underwent a contrast-enhanced examination with a GE3.0T scanner (GEHC MRHDXT). The exclusion criteria were as follows: previous intracranial surgery, radiotherapy, injury, and incomplete preoperative MRI data.

### 2.2. MRI Acquisition

All patients were examined using the same scanner (GE SIGNA 3.0T). Contrast-enhanced T1-weighted imaging (CE-T1WI) was performed in the sagittal, coronal, and axial planes after the intravenous administration of 0.1 mmol/kg Gd-DTPA (field of view, 240 × 240 mm; matrix size, 512 × 512 mm; slice thickness, 5 mm; 90° flip angle; TR, 2250 mm; and TE, 24 ms). All images were digitally stored in a Picture Archiving and Communication Systems (Centricity PACS Radiology RA1000 Workstation, General Electric, Milwaukee, WI, USA) and could be remotely accessed.

### 2.3. MR Image Analysis and Feature Extraction

#### 2.3.1. MR Image Feature Extraction

2D image slices from CE-T1WI of the largest tumor cross-section were loaded into AK software (Analysis Kit, GE Healthcare) for radiomic analysis. Two radiology specialists (NL and LXJ), with 10 and 15 years of experience in medical images, reviewed each patients' images from CE-T1WI independently, and the two reviewers were blinded to the patients' IDH1 mutation status.

**Figure 1:**
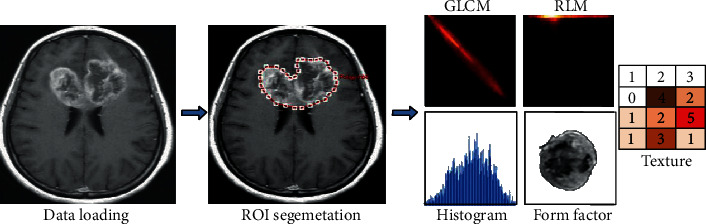
Flow chart of the radiomic analysis of glioma. The original image was imported into GE A.K. (Analysis-Kinetics) analysis software, and the ROI was manually outlined to extract the radiomic features.

Each tumor was manually delineated using the “polygon mode” tool; contrast-enhanced tissue and intratumoral necrosis and cystic degeneration were included in the segmentation, whereas peritumoral edema was excluded. After drawing, the two investigators together reexamined all the segmentations, and modifications were made when both agreed. A total of 396 features were extracted from T1-weighted contrast MR images, including histogram parameters, texture parameters (gray-level cooccurrence matrix (GLCM) parameters, run length matrix (RLM) parameters, and gray-level size zone matrix (GLSZM)), and form factor parameters (Figure 1).

#### 2.3.2. Feature Selection and Radiomic Signature Establishment

Radiomic features were extracted using A.K. software, and feature dimensionality reduction was conducted with the Kruskal-Wallis and Spearman analyses. The least absolute shrinkage and selection operator (Lasso) method was used to select the most useful predictive features from the primary dataset. The radiomic signature (rad score) was calculated for each patient via a linear combination of the selected features weighed by their respective coefficients.

#### 2.3.3. Model Validation

ROC analysis was performed to evaluate the differentiating value of the identified variables, and the sensitivity, specificity, positive predictive value, and negative predictive value were calculated.

#### 2.3.4. Statistical Analysis

All data were recorded in a Microsoft Excel (Microsoft Corp., Redmond, WA) file. Statistical analyses were performed using IBM SPSS version 23.0 (IBM Corp., Armonk, NY) and R statistics software (version 3.5.0, https://www.r-project.org). The age of the IDH1-mutant and IDH1 wild-type glioma patients was tested for normality and homogeneity of variance. The difference between the two groups was compared by an independent sample *t*-test (*P* < 0.05). A Kruskal-Wallis nonparametric test and a Spearman correlation analysis were used to remove redundancy among the radiomic features. The area under the ROC curve was used to evaluate the diagnostic performance of the radiomic model (the diagnostic efficiency of 0.5 < AUC < 0.7 was low, 0.7 < AUC < 0.9 was medium, and 0.9 < AUC was high).

## 3. Results

### 3.1. Clinical Data of the Primary Dataset and the Validation Dataset

The pathological results of all patients were detected by immunohistochemistry, including GFAP, Olig-2, IDH1, S-100, NeuN, and Ki-67. The IDH1 gene type was used as the grouping standard; the result was 79 patients with an IDH1 mutation (45 patients with WHO grade III and 34 patients with WHO grade IV; age range, 24-62 years; mean age, 44 ± 11 years; gender, 49 males and 30 females) and 103 patients with wild-type IDH1 (33 patients with WHO grade III and 70 patients with WHO grade IV; age range, 9-78 years; mean age, 49 ± 16 years; gender, 38 males and 65 females) (Table 1).

There was a significant difference in age between IDH1-mutant and IDH1 wild-type glioma patients (Table 2).

### 3.2. Feature Selection and Radiomic Signature Building

**Figure 2:**
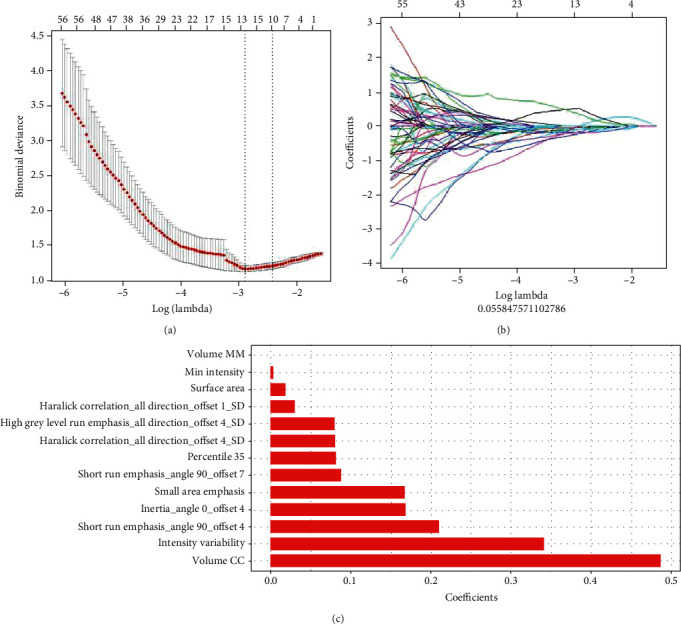
Radiomic feature selection using the Lasso logistic regression model. (a) Selection in the Lasso model used 10-fold cross-validation via minimum criteria to screen the feature set with the best efficiency. (b) A convergence graph of feature coefficients in the Lasso model for feature selection using a 10-fold cross-validation method. (c) Thirteen nonzero coefficients of the radiomic features.

A Lasso logistic regression model was used to screen 396 radiomic features, and 13 nonzero coefficients of radiomic features were obtained. A linear combination of the product of the corresponding weight coefficients was used to form radiomic labels for each patient (Figures 2(a)–2(c)).

### 3.3. Diagnostic Validation of the Radiomic Model

**Figure 3:**
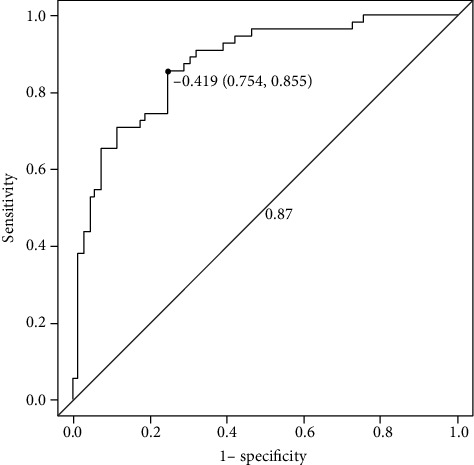
The ROC curve of the primary dataset.

**Figure 4:**
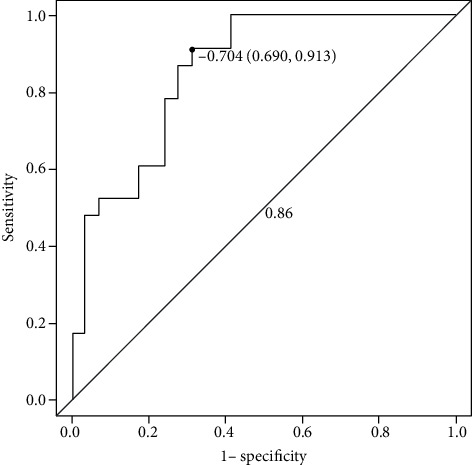
The ROC curve of the validation dataset.

In this study, a biclassification model was established to evaluate IDH1-mutant and IDH1 wild-type gliomas by combining radiomic label features with histopathology. The prediction model showed good discrimination in both the primary dataset (AUC = 0.87, 95% CI: 0.754 to 0.855, ACC = 0.798, sensitivity = 85.5%, specificity = 75.4%, positive predictive value = 0.734, and negative predictive value = 0.867) and the validation dataset (AUC = 0.86, 95% CI: 0.690 to 0.913, ACC = 0.789, sensitivity = 91.3%, specificity = 69.0%, positive predictive value = 0.700, and negative predictive value = 0.909) (Figures 3 and 4, Table 3).

## 4. Discussion

Glioma is the most common primary central nervous system neoplasm in the brain, and strong invasiveness, a poor prognosis, recurrence and aggravation of malignancy, and a high fatality rate comprise its remarkable characteristics. The new WHO classification of central nervous system tumors in 2016 added molecular features to the histological basis and renamed them using histological and molecular features; for example, glioma can be classified into IDH-mutant and wild-type based on the IDH gene mutation. Numerous studies have shown that IDH1 mutations are early molecular changes in diffuse astrocytoma and oligodendroglioma [11], and approximately 70% of grade II-III glioma and secondary glioblastomas harbor an IDH1 mutation [12, 13]. Moreover, studies have shown that IDH1-mutant and wild-type gliomas are different disease entities [14]. IDH1-mutant glioma grows slower than wild-type glioma and is associated with longer overall survival and progression-free survival and with a better prognosis and survival rate [2–9]. This prognostic difference has no significant relationship with the grade of the glioma itself [15]. In addition, studies have shown that [16, 17] an IDH1-specific titanium vaccine can antagonize IDH1-expressing tumor cells and reduce the growth of intracranial tumors. Another line of research [18] has suggested that triptolide can serve as a potent Nrf2 inhibitor, which exhibited selective cytotoxicity to patient-derived IDH1-mutated glioma cells in vitro and in vivo, and can as a valuable therapeutic approach for IDH1-mutated malignancies by targeting the Nrf2-driven glutathione synthesis pathway. Therefore, the preoperative assessment of IDH1 gene mutations in glioma has important clinical predictive value for the diagnosis and prognosis of glioma patients.

In this study, 396 radiomic features were extracted from T1W-enhanced images of 182 patients with glioma. A Lasso logistic regression model was used to screen 13 nonzero coefficients of radiomic features, and the radiomic label and model were established. The prediction model showed good discrimination in both the primary dataset (AUC = 0.87) and the validation dataset (AUC = 0.86). It is concluded that the model has a good predictive effect on differentiating and predicting IDH1-mutant and wild-type gliomas before an operation, and the combination of imaging and genomics can effectively improve the preoperative diagnostic level [19]. In addition, previous study has shown that [20] the radiomic model can accurately predict the IDH1 mutation status of WHO grade II and III glioma by analyzing DTI images. In this study, the IDH1 mutation in WHO grade III and grade IV glioma-enhanced images was studied, which not only confirms the reliability of radiomics in the study of IDH1 mutations in gliomas of different grades but also broadens its application scope. In addition, this study showed that the age of IDH1-mutant glioma patients was younger than that of wild-type glioma patients, and the difference was statistically significant, consistent with previous studies, suggesting that young patients are more likely to suffer from IDH1-mutant glioma and that their postoperative survival and clinical prognosis may be more optimistic.

Our study has some limitations. This study was a retrospective study; data on glioma patients only from our research center were studied, and the model for differentiating IDH-mutant from wild-type glioma needs more research institutes for participation and validation to obtain better promotion and application value. Next, we will conduct more extensive and in-depth research with other research centers to verify the reliability of the model. In addition, some new imaging techniques have been used to predict IDH genotypes in gliomas, such as quantitative imaging of D-2-hydroxyglutarate [21] and MRI-based deep learning method [22]; these noninvasive highly accurate methods for the determination of IDH status can predict IDH status thereby facilitating clinical translation. However, our research is only based on the radiomic model for noninvasive prediction of glioma IDH genotypes, lacking of multiparameter deep learning method. In the next step, we will cooperate with other research centers to carry out multiparameter MRI deep learning method of glioma genotypes, so as to further improve the sensitivity, specificity, and accuracy of preoperative glioma genotype prediction.

In conclusion, the radiomic prediction model based on preoperative-enhanced MR can effectively predict the IDH1 genotype of high-grade glioma before an operation, thus predicting the prognosis and therapeutic effect of glioma, and it can be used as a routine evaluation method before treatment, especially for patients who can undergo only radiotherapy and chemotherapy without surgical resection.

## Figures and Tables

**Table 1:** Clinical data.

Group	Grade		Gender	
	III	IV	M	F
IDH1-mutant (79)	45	34	49	30
Wild-type (103)	33	70	38	65

**Table 2:** Statistical analysis of age in IDH1-mutant and IDH1 wild-type glioma patients.

Group	Age (y)	*T*-test
IDH1-mutant	44 ± 11	*T* = 2.020
IDH1wild-type	49 ± 16	*P* = 0.045

**Table 3:** Diagnostic validation of the radiomic model.

Group	ACC	Sensitivity	Specificity	PPV	NPV
Primary dataset	0.798	0.855	0.754	0.734	0.867
Validation dataset	0.789	0.913	0.690	0.700	0.909

Note: ACC: accuracy; PPV: positive predictive value; NPV: negative predictive value.

## Data Availability

The datasets generated and/or analyzed for the current study are available at the Affiliated Hospital of Qingdao University. The datasets used or analyzed during the current study are available from the corresponding author on reasonable request.
